# The Source of Scarlet Fever

**Published:** 1886-07

**Authors:** 


### Editorial, Etc.

THE SOURCE OF SCARLET FEVER.—As the effects of Scarlet Fever are so often manifested upon teeth, the following will prove interesting:

"A report recently issued by the medical officer of the British local government board presents interesting details of an investigation which appears to have disclosed the original source of scarlet fever, and which, if provisional conclusions should be confirmed by more extended observations, may lead to the extinction of that very destructive disease. That outbreaks of scarlet fever, as of diphtheria, are often connected more or less closely with a particular milk supply, has long been known to persons engaged in sanitary investigations. It has been a familiar observation that cases of scarlet fever were frequent among the customers of certain dairymen, while other consumers, similarly circumstanced but getting their milk from other dealers, seem to enjoy immunity. The theory, however has prevailed that the persons who served the milk were affected by the disease, and when the boy who carried milk to customers was found to have scarcely finished peeling it seemed superfluous to look beyond him for a cause of the spread of the malady. The new view, to which a close study of outbreaks occurring last December in South Marylebone, St. Pancras, Hampstead and Hendon gives strong support, is that the milk itself is the cause of the fever. The districts named, and also St. John's woods, where the scarlet fever appeared at the same time, were supplied from a dairy the sanitary condition of which was excellent. None of the persons employed at it, or in any way connected with it, had had the fever recently, nor had there been a case of scarlet fever in the vicinity for a long time. Although the dairy was indisputably the centre from which the disease was being disseminated, it was impossible to trace any source of human infection. By dint careful inquiry it was ascertained, however, that the commencement of the fever coincided in the point of time with the arrival of four newly-purchased cows, and it was found practicable to connect its ultimate distribution in different districts with the places successively occupied by these cows in different sheds the supplies of milk from which went into hands of different dealers. The four cows on examination turned out to be suffering from an erupted disease of the udders, which produced but little apparent illness, so that they continued to feed satisfactorily and to give plenty of milk. The case against the cows at last became so strong that a dealer among whose customers the fever was prevailing returned his supplies upon the hands of the farmer. The latter ordered the milk to be thrown away, but some of it was obtained from a cowman by poor people near by, with the result that there was an outbreak of scarlet fever among their children a week later. The disease attacked a half dozen families, but none that had not partaken of the rejected milk. The affected cows being placed under observation, it has been found that the erupted disease possesses peculiar characteristics, and that it not only spreads by ordinary contact among animals, but can be communicated to calves by inoculation. Matter obtained from the ulcerations has been cultivated in various media, and particularly in milk, in which it was found to flourish abundantly, producing strings of micrococci possessed of special character. The sub-cultures thus produced being used for inoculation are found to be more virulent than the original virus. Calves were made extremely ill—one was killed—by inoculation with the sub-culture, while but little affected by treatment with virus flesh from the cow. The conjecture is made that in milking a diseased cow pressure upon the udder brings down into the pail infected particles from the sore places left by the eruption, and that the milk into which they fall practically corresponds to an artificial culture of the micrococcus, such as has been found capable of exciting serious and fatal disease when introduced into calves by inoculation. Inoculated calves killed for examination were found to be suffering from inflammatory changes in several vital organs, and especially the kidneys, of a kind absolutely indistinguishable from those that occur in the same organs in the course of human scarlet fever. That. persons taking the strings of micrococci developed in milk into the stomach would have the scarlet fever has not been demonstrated by experiment, through the experience of the families that used the milk that was ordered to be thrown away may be said to point to that conclusion. To boil such milk thoroughly would destroy the scarlet fever micrococcus, if such there be. Science may, however, be invoked to provide us after a time with better means of neutralizing this and other disease germs.

---

